# Extensive Thrombosis in Catastrophic Antiphospholipid Syndrome in a Newly Diagnosed Systemic Lupus Erythematosus: A Case Report

**DOI:** 10.7759/cureus.59542

**Published:** 2024-05-02

**Authors:** Manasawee Tanariyakul, Kevin Edward Nebrejas, Sakditad Saowapa, Natchaya Polpichai

**Affiliations:** 1 Internal Medicine, University of Hawaii John A. Burns School of Medicine, Honolulu, USA; 2 Internal Medicine, Texas Tech University Health Sciences Center, Lubbock, USA; 3 Internal Medicine, Weiss Memorial Hospital, Chicago, USA

**Keywords:** dah, catastrophic aps, acute respiratory distress syndrome [ards], thrombosis, s: sle, microangiopathic hemolytic anemia (maha), peripheral thrombosis, catastrophic antiphospholipid syndrome (caps), systemic lupus erythematosis

## Abstract

In this case report, we present the development of catastrophic antiphospholipid syndrome (CAPS), a rare and potentially fatal consequence of systemic lupus erythematosus (SLE), in a 33-year-old Micronesian woman. CAPS is characterized by extensive arterial thrombosis and multiorgan failure. The patient first showed signs of neuropsychiatric symptoms, brain infarctions on imaging, and severe hypoxic respiratory failure brought into the hospital by diffuse alveolar hemorrhage (DAH) along with lupus nephritis (LN). Blood urea nitrogen (BUN) and creatinine (Cr) were progressively elevated to 102/4.1 mg/dL, respectively. A urinalysis revealed microscopic hematuria and proteinuria with a urine protein/creatinine ratio of 6052 mg/g. She was also found to have had microangiopathic hemolytic anemia (MAHA) and severe venous thrombosis, both of which were indicative of CAPS. An aggressive approach, including immunosuppressive medication, therapeutic plasma exchange, and anticoagulation, had positive results, including renal recovery and the cessation of thrombotic episodes. This instance highlights how crucial it is to identify CAPS patients early and take appropriate action to improve patient outcomes for this difficult and sometimes deadly disorder.

## Introduction

Antiphospholipid syndrome (APS) commonly provokes hypercoagulability, which is caused by antibodies directed against phospholipids and causes thrombosis in veins or arteries. In individuals who tested positive for syphilis serologically, these antibodies were first identified in 1906 [1]. When there is no additional autoimmune illness present, the condition is known as primary. When an autoimmune disease such as systemic lupus erythematosus (SLE) is present, secondary APS might arise.

An uncommon APS consequence, catastrophic antiphospholipid syndrome (CAPS), affects around 1% of patients and is more common in women. This disease's thrombotic episodes may happen in any blood vessel, resulting in a broad range of symptoms. Peripheral thrombosis (deep vein thrombosis in 38.9% of cases) and neurology (20.2% of cases) are linked to the most frequent kinds of presentation [2]. It is characterized by multiorgan failure caused by microvascular thrombosis; the kidney is most often affected, followed by the lungs and the central nervous system (CNS). Only 6-10% of individuals with pulmonary involvement have alveolar bleeding, which is often linked to thrombocytopenia and microangiopathic hemolytic anemia (MAHA) [2]. In this case, we document a case of CAPS in a patient suffering from SLE, emphasizing the therapeutic obstacles and approaches related to this uncommon illness.

This article was previously presented as a meeting abstract at the 2024 Hawaii Chapter of the American College of Physicians (ACP) on March 9, 2024.

## Case presentation

**Figure 1:**
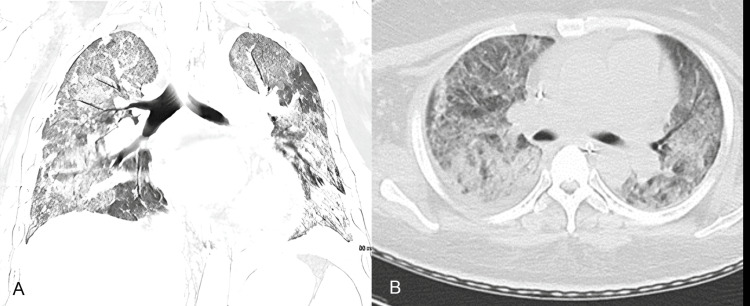
Computed tomography images in the axial plane (A) and coronal plane (B) of the chest, demonstrating bilateral ground glass opacities throughout both lungs. These images represent lung windows on CT scan.

A previously healthy G2P1 33-year-old Micronesian woman with a history of miscarriage at 22 years of age presented to our institution following a recent biopsy-proven diagnosis of class IV lupus nephritis (LN). One week prior to the admission, she exhibited new-onset renal failure, with serum Cr going up from 0.7 to 2.0 within three months. The outpatient nephrologist found her to have elevated anti-dsDNA at 4614 IU/mL, low C3 at 26 mg/dl, and low C4 at less than 2 mg/dl. Additionally, the patient was found to have pancytopenia with a hemoglobin level of 8.1 g/dl, a white blood cell count of 3.0 × 10^9^/L, and a platelet count of 98 × 10^9^/L. A kidney biopsy confirmed class IV lupus nephritis. She was initially treated with mycophenolate mofetil (MMF) 500 mg twice daily, hydroxychloroquine (HCQ) 200 mg daily, and prednisone 40 mg daily. Despite taking the medications for a week, she then developed submassive hemoptysis with acute hypoxic respiratory failure, requiring intubation and mechanical ventilation. She did not have a fever, orthopnea, chest pain, or sputum production previously. Vital signs at presentation included a body temperature of 99.3 °F, a pulse rate of 104 beats per minute, a respiratory rate of 24 per minute, and an elevated blood pressure of 174/100 mmHg. Arterial blood gas measurements on 50% FiO_2_ showed a PaO_2_ of 83 mmHg, indicating a P/F ratio of 166. Chest X-ray and CT scan of the chest demonstrated diffuse bilateral reticulonodular infiltrates without sign of pulmonary thrombosis, and bronchoscopy revealed diffuse alveolar hemorrhage (DAH) (Figure 1a-1b).

Laboratory evaluation revealed acute-to-chronic renal failure with Cr 4.2 mg/dL, hyperkalemia, metabolic acidosis, elevated anti-dsDNA, and low C3 and C4 levels (Table 1). Furthermore, the patient exhibited hemolytic anemia with a blood smear consistent with MAHA. However, the ADAMTS13 level was normal. Sputum culture was found to be negative for bacterial infection. She was started on rituximab 1 g IV and pulse methylprednisolone 1 g IV daily for three days, along with hemodialysis, due to acute renal failure. With an improving respiratory status, the patient was extubated after four days of ventilatory support. However, she subsequently developed neuropsychiatric symptoms, including behavioral changes and visual hallucinations, the day after her extubation. No focal neurological deficit was found on the examination. The MRI brain demonstrated diffuse punctate foci of acute to subacute infarction with narrowing of bilateral anterior cerebral artery (ACA), middle cerebral artery (MCA), and left posterior cerebral artery (PCA) territories (Figure 1). These findings are consistent with SLE with multiple major organ involvements, including diffuse alveolar hemorrhage, lupus cerebritis with stroke and encephalopathy, and lupus nephritis with oliguric renal failure.

**Table 1:** Laboratory findings during the hospitalization H: high, L: low

	Initial presentation	Normal range
Hemoglobin (g/dl)	6.5 (L)	11.2–15.7
Hematocrit (%)	20.6 (L)	34.1–44.9
MCV (fL)	83.4	79.4–98.4
WBC (× 10^3^/μL)	4.93	3.80–10.80
Platelet (× 10^3^/μL)	64	151–424
Prothrombin time (sec)	18.4 (H)	11.8–14.2
Haptoglobin (mg/dL)	<10 (L)	30–200
LDH (IU/L)	750 (H)	135–250
International normalized ratio or INR	1.6	
Partial thromboplastin time (sec)	38.0 (H)	23.5–37.8
BUN (mg/dL)	102 (H)	6–23
Creatinine (mg/dL)	4.1 (H)	0.6–1.4
C3-complement level (mg/dL)	26 (L)	90–180
C4-complement level (mg/dL)	<2 (L)	10–40
Anti-nuclear Ab Titer	1280 (Homogenous pattern) (H)	≤40
Anti-DNA double stranded (IU/mL)	222.0 (H)	≤4
Anti Sm	2.5 (H)	≤0.9
ADAMTS-13 activity (%)	78	40–130
D-dimer (μg/mL)	10.71 (H)	<0.50
Fibrinogen (mg/dl)	154	200–496
Cardiolipin Ab IgA (APL-U/mL)	41.2 (H)	<20.0
Cardiolipin Ab IgG (GPL-U/mL)	63.0 (H)	<20.0
Cardiolipin Ab IgM (MPL-U/mL)	2.8	<20.0
Beta-2 glycoprotein (U/mL)	32.8 (H)	<20.0
Urine, pH	5.5	5.0–7.5
Urine, specific gravity	1.024	1.005–1.030
Urine, protein	3+ (H)	Negative
Urine, RBC (/hpf)	51–100 (H)	0–2
Urine, WBC (/hpf)	6–20 (H)	0–5
Urine, protein/creatinine ratio (<200 mg/g)	6052.3 (H)	<200
Sodium (mEq/L)	145	133–145
Potassium (mEq/L)	5.4 (H)	3.3–5.1
Chloride (mEq/L)	109 (H)	95–108
CO_2_ (mEq/L)	20 (L)	21–30
Arterial pH	7.20 (L)	7.38–7.42
Arterial pCO_2_ (mmHg)	47 (H)	38–42
Arterial pO_2_ (mmHg)	83	80–100
Arterial HCO_3_ (mmol/L)	18 (L)	22–26

A day after her behavioral changes, widespread venous thromboembolism (VTE) further complicated her hospital course. The patient's hemodialysis catheter repeatedly clotted during hemodialysis. The repeated placement of a femoral non-tunneled dialysis catheter was complicated by repeated clotting of the guidewire despite multiple attempts. Subsequent imaging revealed venous thrombosis involving the upper and lower extremities, including the left basilic and cephalic veins, the right common femoral vein, and the left gastrocnemius, popliteal, and femoral veins. A thrombophilia workup was conducted, which was significant for triple-positive antiphospholipid antibodies (anti-cardiolipin, beta-2-glycoprotein, and lupus anticoagulant). Given the multiple organ involvement, intravascular thrombosis, and the presence of antiphospholipid antibodies, suspicion for CAPS was raised. Despite a recent alveolar hemorrhage, the patient was started on a heparin drip for her widespread venous thrombosis, with close observation for recurrent bleeding.

The patient was treated with cyclophosphamide for LN and DAH. Subsequently, her hospitalization was complicated by persistent leukopenia, prompting a switch to rituximab, from which she received a total of four doses: 1 g two days prior to admission, 769 mg in the second week of admission, 769 mg in the third week, and 660 mg in the fourth week. Concurrently, she was managed with a heparin drip, prednisone, and IVIg, and underwent six cycles of plasmapheresis due to suspicion of CAPS. After treatment, her symptoms gradually improved; hemoptysis ceased, and she was found to have recovered neurological, renal, and respiratory functions. Subsequently, treatment included MMF, under which she showed gradual improvement and significant renal recovery, leading to the discontinuation of CRRT. At the time of discharge, her medications were transitioned to include warfarin (targeted to achieve an INR of 2-3), MMF, prednisone, HCQ, and atovaquone (for PJP prophylaxis), with warfarin being prescribed for lifelong use. During her outpatient follow-up four months after discharge, hemolytic anemia resolved and renal function recovered, with Cr levels decreasing to 0.9. Her lupus remained suppressed with prednisone 20 mg daily, HCQ 200 mg daily, and MMF 500 mg BID.

## Discussion

Multiorgan failure is the outcome of diffuse vascular thrombosis in the uncommon and potentially fatal CAPS [3]. The following four requirements must be met for a CAPS diagnosis: three or more organs or tissues must be involved, symptoms must appear within a week, there must be histological proof of vascular thrombosis, and antiphospholipid antibodies must be present [4]. This case illustrates a rare presentation of probable CAPS - meeting three of the four criteria, except for histopathological confirmation - in a patient newly diagnosed with SLE and class IV LN. Her presentation fulfills the new 2023 American College of Rheumatology (ACR) criteria for APS with the presence of at least three clinical domains and two laboratory domains. Clinically, the patient developed manifestations of VTE, intracerebral arterial thrombosis, suspected microvascular complications manifesting as pulmonary hemorrhage, livedo racemosa, and MAHA with thrombocytopenia. Laboratory criteria were met with triple aPL-positivity [5]. A minority of patients can demonstrate more accelerated life-threatening thrombosis and a cataclysmic variant of APS, termed CAPS, using the Asherson criteria. CAPS has a more rapid onset with evidence of multiorgan involvement. The cornerstone of medical management is triple therapy, consisting of anticoagulation, glucocorticoids, therapeutic plasma exchange, and IVIg. Due to the severity of the presentation, the patient received additional immunosuppressive therapy. Prompt recognition and management of underlying CAPS is crucial, given that the mortality rate is as high as 30% [6,7]. 

A differential diagnosis should be considered beyond CAPS in such a complex case. Thrombotic thrombocytopenic purpura (TTP) could be a potential differential, especially given the presence of MAHA and thrombocytopenia; however, the normal ADAMTS13 level makes this less likely. Disseminated intravascular coagulation (DIC) could also be considered due to the widespread thrombosis and hemorrhage along with coagulopathy, but it is typically associated with decreased fibrinogen levels due to a lack of systemic consumption of coagulation factors [8]. Additionally, recent studies have identified celiac disease as a risk factor for both arterial and venous thrombosis due to inflammatory and autoimmune mechanisms that may also lead to hypercoagulability [9]. Given its association with pulmonary complications, such as alveolar hemorrhage and lung hemosiderosis, it could theoretically contribute to or complicate the patient’s respiratory symptoms. However, celiac disease is less commonly linked to extensive organ involvement, and this case lacked a sign of gastrointestinal symptoms or malabsorption.

Intravascular thrombosis, which primarily affects microcirculation and can involve both the arteries and veins in as many as 64% of cases, is the classic presentation of CAPS [9]. A variety of thrombotic events affecting the venous and arterial circulations were evident in our patient, including extensive VTE, cerebral infarctions, DAH, and LN. According to MRI results showing acute to subacute infarcts involving various vascular areas, our patient had neuropsychiatric symptoms typical of cerebral infarctions. Moreover, the development of DAH in our patient emphasizes the pulmonary signs and symptoms of CAPS, which can be brought on by microvascular thrombosis that results in bleeding in the alveoli. Our patient not only had widespread arterial thrombosis but also severe venous thrombosis, affecting both the lower and upper extremities.

About 50% of the time, APS is overlaid with CAPS [3]. Despite not having an APS diagnosis at the time of her current presentation, our patient's history of spontaneous miscarriage while she was 22 years old may have been associated with APS in the past. About 50% of people with CAPS also have another autoimmune condition. SLE accounts for 40% of these, lupus-like disorders for 5%, and other autoimmune illnesses for 9%. Most women who are of reproductive age are affected by CAPS. In most cases, a triggering cause is identifiable. These consist of an infection (22%), surgery (10%), stopping anticoagulation (8%), medicine (7%), a problem during pregnancy (7%), and a malignant process (5%) [3]. The existence of CAPS was verified in our instance by triple-positive antiphospholipid antibodies, highlighting the need for a thorough serological examination in suspected cases.

The most often impacted organs are the kidneys, lungs, heart, brain, liver, and gastrointestinal tract. At the time of presentation, 24% of patients had lung disease, and throughout the course of the illness, 64% of patients developed it [3]. The most frequent pulmonary symptoms are pulmonary embolism and acute respiratory distress syndrome. Arterioles and pulmonary arteries may thrombose sometimes. DAH was found in 6% of cases [10]. It is unclear how DAH in CAPS pathophysiologically develops. The histological study of the lung in APS patients with DAH revealed microvascular thrombosis and, in a few cases, capillaritis. Hemolytic anemia, DAH, respiratory failure, and renal failure were among the signs that our patient had that were consistent with CAPS.

Renal illness eventually affects 71% of individuals with CAPS, but it is only evident in 18% of patients at presentation [3]. Acute thrombotic microangiopathy, which is defined by the deposition of fibrin thrombi in glomeruli, arterioles, or both, is the most frequent finding in histology. Thirty percent of the patients had interstitial inflammation. Microvascular thrombosis is sometimes accompanied by interstitial bleeding [11]. Rarely is immune-complex deposition seen. Chronic kidney damage is indicated by tubular atrophy and interstitial fibrosis. Chronic vascular injury is indicated by the onion skin pattern or concentrated laminations of the fibrotic intima of the arteries and arterioles.

Microangiopathic hemolytic anemia and thrombocytopenia, which are the results of acute thrombotic microangiopathy, are often the causes of the clinical signs of CAPS. The platelet count is less than 100,000/mm in around half of CAPS patients [12]. Antibodies against platelet glycoproteins and glycoprotein-I, which cause platelet activation and aggregation, are thought to be the causes of thrombocytopenia in APS [13]. Notably, when differentiating between DIC and APS, the key aspects to focus on include the fibrinogen levels and the specific presence of antiphospholipid antibodies. DIC is typically acute and associated with systemic consumption coagulopathy, whereas APS is characterized by thrombotic events in the presence of specific antibodies without the broad systemic activation of the coagulation cascade seen in DIC [8].

Other forms of acute thrombotic microangiopathies, such as TTP and hemolytic-uremic syndrome (HUS), may also be related to microangiopathic hemolytic anemia and thrombocytopenia. It might be difficult to distinguish between HUS/TTP and CAPS at times. Schistocytosis is often modest in CAPS and prominent in HUS/TTP. In individuals with TTP, fever and neurologic symptoms often predominate in clinical presentations. Furthermore, the majority of TTP patients have plasma ADAMTS-13 activity that is less than 5% of normal.

High-dose glucocorticoids and anticoagulation are the mainstays of treatment for most CAPS patients [3]. However, other therapeutic measures are often employed in different combinations, including IVIg, cyclophosphamide, plasma exchange, and anticoagulant medications. The death rate has dropped from 53% to 33% as a result of early diagnosis and treatment [14]. In our case, the patient received rituximab, pulse methylprednisolone, heparin, plasmapheresis, and IVIg treatment. Rituximab, a monoclonal antibody targeting the B-cell CD20 antigen, was selected because of its immunomodulatory properties and ability to reduce thrombosis caused by autoimmune processes. Methylprednisolone was administered in pulse form to reduce organ damage and the inflammatory response. To reduce the thrombotic load and eliminate harmful autoantibodies, anticoagulation with heparin and plasmapheresis were started, respectively. Immunoglobulin treatment was also used to neutralize the antibody [15]. Due to the high risk of recurrent disease, lifelong anticoagulant medication with warfarin is essential to lower the risk of VTE.

## Conclusions

Our case report brings attention to the unusual and potentially fatal CAPS associated with SLE. The patient met the diagnostic criteria for CAPS as they had multiorgan failure brought on by extensive arterial thrombosis. A successful result required early diagnosis and vigorous treatment, such as immunosuppressive medication, anticoagulants, and therapeutic plasma exchange. High death rates make CAPS a difficult syndrome to manage, underscoring the need for early detection and treatment. To learn more about the pathogenesis and best practices for treating this uncommon illness, further study is necessary.
